# Supplementary material for: Association of ischemic stroke onset time with presenting severity, acute progression, and long-term outcome: A cohort study
Source: PLoS Med. 2022 Feb 4;19(2):e1003910. doi: 10.1371/journal.pmed.1003910 (PMC8815976; doi:10.1371/journal.pmed.1003910)
Supplement: S1 Table — (DOCX) [file pmed.1003910.s002.docx]

**S1 Table. Baseline characteristics by stroke onset time (4-hour intervals)**

|  | **06:00–10:00 (n=3,927)** | **10:00–14:00 (n=4,642)** | **14:00–18:00 (n=3,880)** | **18:00–22:00 (n=3,159)** | **22:00–02:00 (n=1,060)** | **02:00–06:00 (n=793)** | **p value** |
| --- | --- | --- | --- | --- | --- | --- | --- |
| Age, years | 68.1 (12.7) | 68.0 (13.2) | 67.1 (13.2) | 65.9 (13.7) | 62.5 (14.4) | 65.5 (13.2) | <0.001 |
| Sex, women | 1,507 (38.4) | 1,867 (40.2) | 1,611 (41.5) | 1,244 (39.4) | 398 (37.6) | 273 (34.4) | 0.001 |
| Previous stroke | 813 (20.7) | 1,036 (22.3) | 764 (19.7) | 664 (21.0) | 195 (18.4) | 158 (19.9) | 0.018 |
| Hypertension | 2,539 (64.7) | 3,013 (64.9) | 2,466 (63.6) | 1,991 (63) | 638 (60.2) | 497 (62.7) | 0.05 |
| Diabetes | 1,042 (26.5) | 1,419 (30.6) | 1,072 (27.6) | 857 (27.1) | 298 (28.1) | 228 (28.8) | 0.001 |
| Hyperlipidemia | 1,181 (30.1) | 1,514 (32.6) | 1,189 (30.6) | 1,013 (32.1) | 371 (35.0) | 216 (27.2) | 0.001 |
| Current or recent^*^ smoking | 1,393 (35.5) | 1,591 (34.3) | 1,365 (35.2) | 1,160 (36.7) | 435 (41.0) | 314 (39.6) | <0.001 |
| Atrial fibrillation | 959 (24.4) | 1,168 (25.2) | 1,012 (26.1) | 861 (27–3) | 266 (25.1) | 193 (24.3) | 0.10 |
| Coronary artery disease | 361 (9.2) | 467 (10.1) | 348 (9.0) | 318 (10.1) | 101 (9.5) | 78 (9.8) | 0.48 |
| Prestroke mRS 0 or 1 | 3,533 (90.0) | 4,184 (90.1) | 3,512 (90.5) | 2,859 (90.5) | 977 (92.2) | 710 (89.5) | 0.33 |
| Prestroke antiplatelet use | 1,115 (28.4) | 1,411 (30.4) | 1,097 (28.3) | 881 (27.9) | 279 (26.3) | 221 (27.9) | 0.046 |
| Prestroke statin use | 826 (21.0) | 1,058 (22.8) | 774 (19.9) | 673 (21.3) | 250 (23.6) | 153 (19.3) | 0.008 |
| Prestroke antihypertensive use | 2,049 (52.2) | 2,392 (51.5) | 1,888 (48.7) | 1,542 (48.8) | 495 (46.7) | 382 (48.2) | 0.001 |
| Prestroke antidiabetic use | 775 (19.7) | 1,067 (23.0) | 808 (20.8) | 630 (19.9) | 218 (20.6) | 184 (23.2) | 0.002 |
| Admission NIHSS score | 3 (1 to 9) | 3 (1 to 9) | 3 (1 to 9) | 3 (1 to 9) | 3 (1 to 8) | 4 (1 to 10) | 0.005 |
| Time from onset to hospital arrival, hour | 2.2 (1.0 to 3.7) | 1.9 (0.9 to 3.4) | 1.7 (0.8 to 3.2) | 1.4 (0.8 to 2.6) | 1.2 (0.6 to 2.2) | 2.1 (1.0 to 4.0) | <0.001^†^ |
| Stroke subtype |  |  |  |  |  |  | <0.001 |
| Large artery atherosclerosis | 1,047 (26.7) | 1,254 (27.0) | 949 (24.5) | 731 (23.1) | 268 (25.3) | 218 (27.5) |  |
| Small vessel occlusion | 438 (11.2) | 493 (10.6) | 423 (10.9) | 339 (10.7) | 131 (12.4) | 99 (12.5) |  |
| Cardioembolism | 970 (24.7) | 1,145 (24.7) | 982 (25.3) | 825 (26.1) | 252 (23.8) | 193 (24.3) |  |
| Undetermined | 875 (22.3) | 978 (21.1) | 861 (22.2) | 634 (20.1) | 200 (18.9) | 181 (22.8) |  |
| Other-determined | 110 (2.8) | 133 (2.9) | 112 (2.9) | 84 (2.7) | 39 (3.7) | 16 (2.0) |  |
| Transient ischemic attack | 487 (12.4) | 639 (13.8) | 553 (14.3) | 546 (17.3) | 170 (16.0) | 86 (10.8) | <0.001 |
| Revascularization therapy | 1,325 (33.7) | 1,560 (33.6) | 1,354 (34.9) | 1,150 (36.4) | 372 (35.1) | 290 (36.6) | 0.09 |
| Intravenous | 777 (19.8) | 959 (20.7) | 844 (21.8) | 769 (24.3) | 271 (25.6) | 172 (21.7) |  |
| Intra-arterial | 214 (5.4) | 219 (4.7) | 159 (4.1) | 127 (4.0) | 22 (2.1) | 59 (7.4) |  |
| Intravenous + intra-arterial | 334 (8.5) | 382 (8.2) | 351 (9.0) | 254 (8.0) | 79 (7.5) | 59 (7.4) |  |
| Door-to-needle time, min | 35 (26 to 48) | 37 (27 to 50) | 37 (27 to 49) | 36 (27 to 52) | 40 (28 to 52) | 40 (30 to 53) | 0.002^†^ |
| Door-to-puncture time, min | 97 (72 to 126) | 94 (72 to 125) | 104 (80 to 136) | 109 (85 to 139) | 125 (95 to 156) | 120 (93 to 150) | <0.001^†^ |
| Seasons |  |  |  |  |  |  | 0.35 |
| Spring | 995 (25.3) | 1,136 (24.5) | 990 (25.5) | 775 (24.5) | 273 (25.8) | 194 (24.5) |  |
| Summer | 1,026 (26.1) | 1,246 (26.8) | 982 (25.3) | 851 (26.9) | 294 (27.7) | 226 (28.5) |  |
| Fall | 1,018 (25.9) | 1,151 (24.8) | 990 (25.5) | 763 (24.2) | 235 (22.2) | 201 (25.4) |  |
| Winter | 888 (22.6) | 1,109 (23.9) | 918 (23.7) | 770 (24.4) | 258 (24.3) | 172 (21.7) |  |

Data are mean (SD), number (%), or median (interquartile range). Analysis of variance and chi-square test were used. mRS=modified Rankin Scale. NIHSS=National Institutes of Health Stroke scale. IQR=interquartile range. ^*^Quit smoking within 5 years of stroke onset. ^†^Kruskal-Wallis test was used
