# Supplementary material for: Association of ischemic stroke onset time with presenting severity, acute progression, and long-term outcome: A cohort study
Source: PLoS Med. 2022 Feb 4;19(2):e1003910. doi: 10.1371/journal.pmed.1003910 (PMC8815976; doi:10.1371/journal.pmed.1003910)
Supplement: S2 Table — NIHSS, National Institutes of Health Stroke Scale. (DOCX) [file pmed.1003910.s003.docx]

**S2 Table. Mixed-effects negative binomial logistic regression analysis between admission NIHSS score and stroke onset time**

|  | 06:00–10:00 | 10:00–14:00 | 14:00–18:00 | 18:00–22:00 | 22:00–02:00 | 02:00–06:00 |
| --- | --- | --- | --- | --- | --- | --- |
| **All patients** |  |  |  |  |  |  |
| Number of patients | 3,927 | 4,642 | 3,880 | 3,159 | 1,060 | 793 |
| Estimated mean NIHSS (95% CI) | 5.7 (5.3 to 6.0) | 5.7 (5.4 to 6.0) | 5.7 (5.4 to 6.0) | 5.8 (5.5 to 6.2) | 6.1 (5.6 to 6.6) | 6.6 (6.0 to 7.1) |
| Adjusted mean difference (95% CI) | Reference | 0.1 (–0.2 to 0.3) | 0.0 (–0.2 to 0.3) | 0.2 (–0.1 to 0.5) | 0.4 (0.0 to 0.9) | 0.9 (0.4 to 1.4) |
| p value | Reference | 0.66 | 0.71 | 0.20 | 0.04 | < 0.001 |
| **Large artery atherosclerosis** |  |  |  |  |  |  |
| Number of patients | 1,047 | 1,254 | 949 | 731 | 268 | 218 |
| Estimated mean NIHSS (95% CI) | 5.0 (4.6 to 5.4) | 4.8 (4.5 to 5.2) | 5.2 (4.8 to 5.6) | 5.2 (4.8 to 5.7) | 5.3 (4.6 to 6.0) | 5.9 (5.1 to 6.7) |
| Adjusted mean difference (95% CI) | Reference | –0.2 (–0.6 to 0.2) | 0.2 (–0.2 to 0.6) | 0.2 (–0.3 to 0.7) | 0.3 (–0.4 to 1.0) | 0.9 (0.1 to 1.7) |
| p value | Reference | 0.31 | 0.39 | 0.40 | 0.38 | 0.023 |
| **Small vessel occlusion** |  |  |  |  |  |  |
| Number of patients | 438 | 493 | 423 | 339 | 131 | 99 |
| Estimated mean NIHSS (95% CI) | 3.0 (2.7 to 3.2) | 2.8 (2.6 to 3.1) | 2.6 (2.4 to 2.9) | 2.8 (2.5 to 3.1) | 3.4 (2.9 to 3.9) | 3.0 (2.5 to 3.5) |
| Adjusted mean difference (95% CI) | Reference | –0.1 (–0.4 to 0.2) | –0.3 (–0.6 to 0.0) | –0.1 (–0.4 to 0.2) | 0.5 (–0.1 to 1.0) | 0.0 (–0.5 to 0.5) |
| p value | Reference | 0.41 | 0.048 | 0.54 | 0.069 | 0.96 |
| **Cardioembolism** |  |  |  |  |  |  |
| Number of patients | 970 | 1,145 | 982 | 825 | 252 | 193 |
| Estimated mean NIHSS (95% CI) | 9.1 (8.4 to 9.7) | 9.9 (9.3 to 10.6) | 9.7 (9.0 to 10.3) | 9.9 (9.2 to 10.6) | 10.3 (9.1 to 11.4) | 10.7 (9.3 to 12.1) |
| Adjusted mean difference (95% CI) | Reference | 0.9 (0.2 to 1.5) | 0.6 (–0.1 to 1.3) | 0.8 (0.1 to 1.6) | 1.2 (0.0 to 2.4) | 1.6 (0.3 to 3.0) |
| p value | Reference | 0.014 | 0.09 | 0.03 | 0.035 | 0.012 |

NIHSS=National Institutes of Health Stroke Scale. CI=confidence interval. Mixed-effects negative binomial regression was used with adjustment for age, sex, prestroke modified Rankin Scale score, previous stroke, hypertension, diabetes, hyperlipidemia, atrial fibrillation, smoking, stroke subtype, time from onset to hospital arrival, prestroke antiplatelet use, season of stroke onset, and prestroke statin use.
