# Supplementary material for: Association of ischemic stroke onset time with presenting severity, acute progression, and long-term outcome: A cohort study
Source: PLoS Med. 2022 Feb 4;19(2):e1003910. doi: 10.1371/journal.pmed.1003910 (PMC8815976; doi:10.1371/journal.pmed.1003910)
Supplement: S3 Table — NIHSS, National Institutes of Health Stroke Scale. (DOCX) [file pmed.1003910.s004.docx]

**S3 Table****. Unadjusted associations of stroke onset time (4-hour intervals) with admission NIHSS score, neurological deterioration, and 3-month functional outcome after stratification with stroke subtypes**

|  | 06:00–10:00 | 10:00–14:00 | 14:00–18:00 | 18:00–22:00 | 22:00–02:00 | 02:00–06:00 |
| --- | --- | --- | --- | --- | --- | --- |
| **Large artery atherosclerosis** |  |  |  |  |  |  |
| Number of patients | 1,047 | 1,254 | 949 | 731 | 268 | 218 |
| Admission NIHSS score^*^ |  |  |  |  |  |  |
| Unadjusted common odds ratio (95% CI) | Reference | 0.96 (0.82 to 1.12) | 1.08 (0.92 to 1.27) | 1.05 (0.88 to 1.25) | 1.12 (0.88 to 1.43) | 1.41 (1.08 to 1.85) |
| p value | Reference | 0.59 | 0.34 | 0.60 | 0.35 | 0.012 |
| Early neurological deterioration |  |  |  |  |  |  |
| Unadjusted incidence, n (%) | 158 (15.1) | 231 (18.4) | 146 (15.4) | 149 (20.4) | 57 (21.3) | 38 (17.4) |
| Unadjusted risk difference (95% CI), % | Reference | 3.3 (0.3 to 6.4) | 0.3 (–2.9 to 3.5) | 5.3 (1.7 to 8.9) | 6.2 (0.8 to 11.5) | 2.3 (–3.1 to 7.8) |
| p value | Reference | 0.03 | 0.87 | 0.004 | 0.02 | 0.39 |
| Favorable outcome^†^ |  |  |  |  |  |  |
| Unadjusted incidence, n (%) | 668 (77.1) | 803 (78.2) | 621 (77.4) | 481 (77.5) | 164 (75.9) | 126 (70.0) |
| Unadjusted risk difference (95% CI), % | Reference | 1.1 (–2.7 to 4.8) | 0.3 (–3.7 to 4.3) | 0.4 (–4.0 to 4.6) | –1.2 (–7.6 to 5.1) | –7.1 (–14.4 to 0.1) |
| p value | Reference | 0.58 | 0.89 | 0.89 | 0.71 | 0.054 |
| **Small vessel occlusion** |  |  |  |  |  |  |
| Number of patients | 438 | 493 | 423 | 339 | 131 | 99 |
| Admission NIHSS score^*^ |  |  |  |  |  |  |
| Unadjusted common odds ratio (95% CI) | Reference | 0.85 (0.66 to 1.09) | 0.85 (0.66 to 1.11) | 0.95 (0.72 to 1.25) | 1.22 (0.83 to 1.79) | 0.98 (0.64 to 1.51) |
| p value | Reference | 0.19 | 0.24 | 0.70 | 0.32 | 0.93 |
| Early neurological deterioration |  |  |  |  |  |  |
| Unadjusted incidence, n (%) | 57 (13.0) | 63 (12.8) | 45 (10.6) | 49 (14.5) | 16 (12.2) | 18 (18.2) |
| Unadjusted risk difference (95% CI), % | Reference | –0.2 (–4.5 to 4.1) | –2.4 (–6.7 to 1.9) | 1.4 (–3.5 to 6.3) | –0.8 (–7.2 to 5.6) | 5.2 (–3.1 to 13.4) |
| p value | Reference | 0.92 | 0.28 | 0.56 | 0.81 | 0.22 |
| Favorable outcome^†^ |  |  |  |  |  |  |
| Unadjusted incidence, n (%) | 354 (88.9) | 399 (92.4) | 353 (93.1) | 272 (93.8) | 105 (96.3) | 79 (92.9) |
| Unadjusted risk difference (95% CI), % | Reference | 3.4 (–0.6 to 7.4) | 4.2 (0.2 to 8.2) | 4.8 (0.7 to 9.0) | 7.4 (2.7 to 12.1) | 4.0 (–2.3 to 10.3) |
| p value | Reference | 0.09 | 0.04 | 0.02 | 0.002 | 0.21 |
| **Cardioembolism** |  |  |  |  |  |  |
| Number of patients | 970 | 1,145 | 982 | 825 | 252 | 193 |
| Admission NIHSS score^*^ |  |  |  |  |  |  |
| Common odds ratios (95% CI) | Reference | 1.24 (1.05 to 1.47) | 1.30 (1.09 to 1.54) | 1.21 (1.01 to 1.45) | 1.21 (0.93 to 1.59) | 1.36 (1.01 to 1.84) |
| p value | Reference | 0.009 | 0.003 | 0.034 | 0.15 | 0.043 |
| Early neurological deterioration |  |  |  |  |  |  |
| Unadjusted incidence, n (%) | 131 (13.5) | 169 (14.8) | 138 (14.1) | 115 (13.9) | 33 (13.1) | 27 (14.0) |
| Unadjusted risk difference (95% CI), % | Reference | 1.3 (–1.7 to 4.2) | 0.5 (–2.5 to 3.6) | 0.4 (–2.8 to 3.6) | –0.4 (–5.1 to 4.3) | 0.5 (–4.9 to 5.8) |
| p value | Reference | 0.41 | 0.73 | 0.79 | 0.86 | 0.86 |
| Favorable outcome^†^ |  |  |  |  |  |  |
| Unadjusted incidence, n (%) | 508 (60.8) | 578 (57.4) | 532 (61.3) | 432 (58.9) | 133 (61.6) | 92 (54.4) |
| Unadjusted risk difference (95% CI), % | Reference | –3.4 (–7.9 to 1.1) | 0.5 (–4.2 to 5.1) | –2.0 (–6.8 to 2.9) | 0.7 (–6.5 to 8.0) | –6.4 (–14.6 to 1.8) |
| p value | Reference | 0.13 | 0.85 | 0.42 | 0.84 | 0.13 |

NIHSS=National Institutes of Health Stroke Scale; CI=confidence interval. ^*^Admission NIHSS score were categorized into three groups (0–1, 2–6, and ≥7). Mixed-effects ordered logistic regression was used. ^†^3-month modified Rankin scale score 0–2 versus 3–6 (unfavorable).
