# Supplementary material for: Association of ischemic stroke onset time with presenting severity, acute progression, and long-term outcome: A cohort study
Source: PLoS Med. 2022 Feb 4;19(2):e1003910. doi: 10.1371/journal.pmed.1003910 (PMC8815976; doi:10.1371/journal.pmed.1003910)
Supplement: S5 Table — END, early neurological deterioration; TIA, transient ischemic attack. (DOCX) [file pmed.1003910.s006.docx]

**S5 Table. Multivariable associations between stroke onset time and END after exclusion of TIA patients**

| **Stroke onset time** | **Unadjusted END, n (%)** | **Adjusted^*^ END, % (95% CI)** | **Adjusted risk difference**^†^**, % (95% CI)** | **p value** |
| --- | --- | --- | --- | --- |
| 06:00–18:00 | 1,527 (14.2%) | 14.7 (13.3 to 16.0) | Reference |  |
| 18:00–06:00 | 656 (15.6%) | 16.4 (14.7 to 18.0) | 1.7 (0.4 to 3.0) | 0.012 |

TIA=transient ischemic attack; END=early neurological deterioration; CI=confidence interval. ^*^Mixed-effects logistic regression with an interaction term between stroke onset time and weekdays (versus weekend) was used. Multivariable adjustment for age, sex, prestroke modified Rankin Scale score, National Institutes of Health Stroke Scale score, previous stroke, hypertension, diabetes, hyperlipidemia, atrial fibrillation, smoking, stroke subtype, time from onset to hospital arrival, prestroke antiplatelet use, season of stroke onset, and prestroke statin use. ^†^Risk differences are for night-onset strokes relative to day-onset strokes.
