# Supplementary material for: Association of ischemic stroke onset time with presenting severity, acute progression, and long-term outcome: A cohort study
Source: PLoS Med. 2022 Feb 4;19(2):e1003910. doi: 10.1371/journal.pmed.1003910 (PMC8815976; doi:10.1371/journal.pmed.1003910)
Supplement: S2 Fig — END, early neurological deterioration. (DOCX) [file pmed.1003910.s008.docx]

**S2 Figure. Adjusted incidence of early neurological deterioration stratified by stroke onset time and stroke subtype**

Error bar indicates 95% confidence interval. Multivariable adjustment for age, sex, prestroke modified Rankin Scale score, admission National Institutes of Health Stroke Scale score, previous stroke, hypertension, diabetes, hyperlipidemia, atrial fibrillation, smoking, time from onset to hospital arrival, prestroke antiplatelet use, season of stroke onset and prestroke statin use. p for interaction by stroke subtype=0.09.
