# Supplementary material for: Association of ischemic stroke onset time with presenting severity, acute progression, and long-term outcome: A cohort study
Source: PLoS Med. 2022 Feb 4;19(2):e1003910. doi: 10.1371/journal.pmed.1003910 (PMC8815976; doi:10.1371/journal.pmed.1003910)
Supplement: S3 Fig — END, early neurological deterioration; NIHSS, National Institutes of Health Stroke Scale; SITS-MOST, Safe Implementation of Thrombolysis in Stroke-Monitoring Study. (DOCX) [file pmed.1003910.s009.docx]

**S3 Figure. Multivariable associations between stroke onset time and early neurological deterioration defined by the Safe Implementation of Thrombolysis in Stroke-Monitoring Study (SITS-MOST) criteria^*^**

Error bar indicates 95% confidence interval. Multivariable adjustment for age, sex, prestroke modified Rankin Scale score, admission National Institutes of Health Stroke Scale (NIHSS) score, previous stroke, hypertension, diabetes, hyperlipidemia, atrial fibrillation, smoking, stroke subtype, time from onset to hospital arrival, prestroke antiplatelet use, season of stroke onset and prestroke statin use. ^*^Early neurological deterioration was defined as an increase of total NIHSS score of 4 or more compared with baseline NIHSS or the lowest NIHSS value.
