# Supplementary material for: Association of ischemic stroke onset time with presenting severity, acute progression, and long-term outcome: A cohort study
Source: PLoS Med. 2022 Feb 4;19(2):e1003910. doi: 10.1371/journal.pmed.1003910 (PMC8815976; doi:10.1371/journal.pmed.1003910)
Supplement: S1 Text — (DOCX) [file pmed.1003910.s010.docx]

**S1 Text. List of institutional review boards**

Dongguk University Ilsan Hospital, Goyang, Korea

Inje University Ilsan Paik Hospital, Goyang, Korea

Seoul National University Bundang Hospital, Seongnam, Korea

Chonnam National University Hospital, Gwangju, Korea

Soonchunhyang University Hospital, Seoul, Korea

Seoul Medical Center, Seoul, Korea

Nowon Eulji Medical Center, Eulji University School of Medicine, Seoul, Korea

Hallym University Sacred Heart Hospital, Anyang, Korea

Eulji University Hospital, Daejeon, Korea

Dong-A University Hospital, Busan, Korea

Yeungnam University Hospital, Daegu, Korea
