# Supplementary material for: Association of ischemic stroke onset time with presenting severity, acute progression, and long-term outcome: A cohort study
Source: PLoS Med. 2022 Feb 4;19(2):e1003910. doi: 10.1371/journal.pmed.1003910 (PMC8815976; doi:10.1371/journal.pmed.1003910)
Supplement: S2 Text — (DOCX) [file pmed.1003910.s011.docx]

**S2 Analysis plan: Association between circadian onset and ischemic stroke outcome**

**Final title**

**Association of ischemic stroke onset time with presenting severity, acute progression, and long-term outcome: A cohort study**

- **Research Questions**

Is circadian stroke onset associated with stroke outcomes?

Specifically, is the night-onset stroke associated with more severe stroke, more frequent early neurological deterioration and worse 3-month functional outcome?

- **Study population**

This study will be conducted using a prospective multicenter stroke registry: Clinical Research Collaboration for Stroke-Korea (CRCS-K). Using a standardized protocol, data will be collected from all patients with acute ischemic stroke or TIA who were admitted to 11 academic hospitals within 7 days of symptom onset between May 2011 and July 2020. Inclusion criteria for this study were: 1) witnessed stroke onset, 2) hospital arrival within 6 hours of onset, and 3) consent to be monitored for the CRCS-K registry-related post-stroke outcomes.

Exclusion criteria

(1) refusal to give research consent to being monitored for stroke outcomes; (2) unwitnessed stroke; and (3) arrival to the participating center after 6 hours of onset.

- **Outcomes**

Stroke severity (National Institute of Health Stroke Scale; NIHSS), early neurological deterioration, and 3-month modified Rankin Scale scores.

NIHSS score at admission

Early neurological deterioration:

1. an increment in total NIHSS score of ≥2 points
2. an increment in NIHSS consciousness score (1a–1c) of ≥1
3. an increment in NIHSS motor score (5a–6b) ≥1
4. any new neurological deficit not assessed by the NIHSS

3-month modified Rankin Scale score:

0: No symptoms at all

1: No significant disability despite symptoms; able to carry out all usual duties and activities

2: Slight disability; unable to carry out all previous activities, but able to look after own affairs without assistance

3: Moderate disability; requiring some help, but able to walk without assistance

4: Moderately severe disability; unable to walk without assistance and unable to attend to own bodily needs without assistance

5: Severe disability; bedridden, incontinent and requiring constant nursing care and attention

6: Dead

- **Covariates**

Information on the following covariates will be collected from this prospective stroke registry: demographic data, medication history, and details regarding vascular risk factors.

Covariates for statistical analysis: age, sex, pre-stroke mRS score, admission NIHSS score, previous stroke, hypertension, diabetes, hyperlipidemia, atrial fibrillation, smoking, stroke subtype, time from onset to hospital arrival, pre-stroke antiplatelet use, and pre-stroke statin use

- **Statistical analysis**
- Group: Analyses will comprehensively evaluate circadian effects comparing both night-onset (18:00–06:00) vs day-onset (06:00–18:00) strokes and at a more granular level with stratification by 4-hour time intervals.
- Main analysis
  - Mixed-effects logistic regression models: associations between stroke onset time and early neurological deterioration while accounting for clustering by hospitals.
  - Mixed-effects negative binomial regression with log link: effect of stroke onset time on admission NIHSS scores
  - Mixed-effects ordered logistic regression models: association between stroke onset time and 3-month modified Rankin Scale scores 🡪 if the proportional odds assumption is violated, 3-month modified Rankin scale score will be dichotomized into two groups (3-month modified Rankin Scale score 0–2 [favorable] vs 3–6 [unfavorable]) and a mixed-effects logistic regression model with adjustment for the same covariates in the models for stroke onset time vs early neurological deterioration will be applied. Stroke etiology-related differences in neurological severity, pathophysiological mechanisms, and circadian variation will be explored using statistical analyses after stratification by three stroke subtypes. The modifying effects of stroke subtypes will be examined by entering an interaction term between stroke onset time and stroke subtypes into the model.
- Sensitivity analyses
  - Will be performed using the early neurological deterioration criteria of the Safe Implementation of Thrombolysis in Stroke-Monitoring Study (SITS-MOST, increase in total NIHSS score ≥4).

***Note:***

*During the study, we modified or added some statical analyses, which was based on suggestions or requests by authors, editors, and reviewers:*

- *A co-author suggested investigating ‘effect modification by weekdays vs. weekend to examine’ to see whether study results were confounded by changes in provision of care during off-hours.*
- *During the review and revision process, one of the reviewers suggested to perform statistical analysis, where admission NIHSS was not handled as a continuous variable: we thus categorized NIHSS scores into three groups (0–1, 2–6, and ≥ 7)* *with a similar number of patients in each group and performed mixed-effects ordered logistic regression analysis.*
- *As suggested by both reviewers, we included stroke onset seasons as an additional covariate.*
- *One of the reviewers suggested to perform an additional sensitivity analysis after excluding patients with transient ischemic attack: we carried out mixed-effects logistic regression analysis between stroke onset time and early neurological deterioration after excluding the patients with transient ischemic attack.*
