# Supplementary material for: Association of ischemic stroke onset time with presenting severity, acute progression, and long-term outcome: A cohort study
Source: PLoS Med. 2022 Feb 4;19(2):e1003910. doi: 10.1371/journal.pmed.1003910 (PMC8815976; doi:10.1371/journal.pmed.1003910)
Supplement: Attachment — Submitted filename: ryu.pdf [file pmed.1003910.s012.pdf]

This paper reports a cohort study of the association between timing of stroke onset and outcomes.

I was asked for a statistical report and I interpret that to include all aspects of the design and conduct of the study.

Points of detail

Page 5 If some of the measurements are observations not planned in the protocol does this possibly introduce a bias?

Page 5 It might be helpful to give the composition of the adjudication committee at least in terms of professional representation. I assume they did not adjudicate patients for whose clinical care they had been responsible?

Page 6 The authors have used two different ways of dividing up the day. Categorising an essentially continuous variable wastes information (Altman and Royston, 2006; Royston et al., 2006) and leads to models which are often implausible as they predict the effect remaining flat within categories and then jumping to a new value at the category boundary. In this case is there an agreed convention for the 0600–1800 versus 1800–0600 split? There is plenty of data so I wonder whether even narrower categories than four–hourly would be better to show the form of relationship.

Page 6 Functional outcome is an ordered categorical variable. Did the authors not consider using ordered logistic regression here?

Page 7 Some of the variables referred to here and displayed in Table 1 do not seem to me to be baseline characteristics. For example revascularisation, END, favourable outcome.

Page 9 Since the NIHSS score is presumably skewed why not just present the exponentiated coefficient as a percentage increase with its confidence interval rather than two means?

Page 10 As someone with a limited knowledge of acute stroke care I struggle to understand why revascularised patients do so much worse.

Page 11, Figure 2 This is where I think finer categories might be helpful. I would be cautious anyway about comparing one category with another in statistical significance when it is really the shape which matters.

Summary

Mostly point for clarification. No major issues for me.

Michael Dewey

References

D G Altman and P Royston. The cost of dichotomising continuous variables. *British Medical Journal*, 332:1080, 2006.

P Royston, D G Altman, and W Sauerbrei. Dichotomizing continuous predictors in multiple regression: a bad idea. *Statistics in Medicine*, 25:127–141, 2006.
