# Supplementary material for: Association of ischemic stroke onset time with presenting severity, acute progression, and long-term outcome: A cohort study
Source: PLoS Med. 2022 Feb 4;19(2):e1003910. doi: 10.1371/journal.pmed.1003910 (PMC8815976; doi:10.1371/journal.pmed.1003910)
Supplement: Attachment — Submitted filename: Response_to_reviewers_Final.docx [file pmed.1003910.s013.docx]

**PMEDICINE-D-21-04039R1**

Circadian Time of Ischemic Stroke Onset Affects Presenting Severity, Acute Progression, and Long-term Outcome

**New Title**

Association of circadian time of ischemic stroke onset with presenting severity, acute progression, and long-term outcome: A cohort study

2021-11-17

**Dear Editor**

We thank you and reviewers of ‘PLOS Medicine’ for taking time to review our manuscript and giving us a chance to revise the paper. We have tried our best to follow all editorial comments and reviewer suggestions, and performed additional analyses, and made corrections and clarifications, highlighted in yellow, in the manuscript. We were deeply impressed with and grateful for the very constructive review, which led us to substantially improve the quality of the manuscript.

**Editorial Comments:**

**Comment 1**

To facilitate review, please include continuous line numbering in the margin of your manuscript.

**Reply 1**

In the revised manuscript, we have included continuous line numbering.

**Comment 2**

Please revise your title according to PLOS Medicine's style. Your title must be nondeclarative and not a question. It should begin with main concept if possible. "Effect of" should be used only if causality can be inferred, i.e., for an RCT. Please place the study design (“a cohort study”) in the subtitle (ie, after a colon).

**Reply 2**

We have changed the title to “Association of circadian time of ischemic stroke onset with presenting severity, acute progression, and long-term outcome: A cohort study”.

**Comment 3**

Please remove the ‘Funding’ information from your acknowledgements section and instead please enter this into the submission form under the ‘Financial Disclosure’ section.

**Reply 3**

We have removed the ‘Funding information’ in ‘Acknowledgements’ section.

**Comment 4**

The Data Availability Statement (DAS) requires revision. If the data are owned by a third party but freely available upon request, please note this and state the owner of the data set and contact information for data requests (web or email address). Note that a study author cannot be the contact person for the data.

**Reply 4**

We have revised the Data Availability Statement as follows:

Data Availability Statement: The informed consent obtained from the study participants does not allow the data to be made freely available through any third party maintained public repository. However, data used for this submission can be made available upon reasonable request (contact details can be found at http://www.strokedb.or.kr/) and the approval of the ‘Comprehensive Registry Collaboration for Stroke in Korea’ steering committee.

**Comment 5**

Abstract Methods and Findings:
* Please ensure that all numbers presented in the abstract are present and identical to numbers presented in the main manuscript text.
* Please include the setting, year in which the study took place, length of follow up, and main outcome measures.
* Please include the important dependent variables that are adjusted for in the analyses.
* In the last sentence of the Abstract Methods and Findings section, please describe the main limitation(s) of the study's methodology.

**Reply 5**

We have revised the manuscript in accordance with the comments.

**Comment 6**

At this stage, we ask that you include a short, non-technical Author Summary of your research to make findings accessible to a wide audience that includes both scientists and non-scientists. The Author Summary should immediately follow the Abstract in your revised manuscript. This text is subject to editorial change and should be distinct from the scientific abstract. Please see our author guidelines for more information: <https://journals.plos.org/plosmedicine/s/revising-your-manuscript#loc-author-summary>

**Reply 6**

We have included the ‘Author Summary’, as follows:

Author Summary

Why was this study done?

- While multiple studies investigated circadian variation in timing of stroke onset and provision of stroke therapies, only one study was found evaluating presenting stroke severity. A recent pooled analysis of CT perfusion imaging data from in 583 patients with anterior large-vessel occlusion stroke found larger initial ischaemic core volume (median, 40.2mL *vs* 33.8mL) and faster core growth progression from onset to imaging with night-onset compared with day-onset stroke. No study evaluated circadian rhythm variation in extent of neurologic deficits, frequency of early neurologic deterioration, or long-term outcome.

What did the researchers do and find?

- In this analysis of multicenter data on 17461 consecutive patients with ischemic stroke presenting within 6 hours of witnessed onset, we found circadian variation in clinical characteristics and course. Night-onset stroke patients presented with greater deficit severity, had a higher likelihood of experiencing early neurologic deterioration during the first 72 hours after symptom onset, and a lower likelihood of favourable 3-month global disability outcome than day-onset stroke patients.
- Circadian variation patterns differed among ischemic stroke subtypes in a manner suggesting modulation by collateral flow availability, with large artery atherosclerosis patients showing more frequent early neurologic deterioration and worse 3-month functional outcome with night-onset, cardioembolic stroke patients showing more severe presenting deficits and worse 3-month functional outcome with night-onset, and small vessel disease patients not evidencing night-day differences.

What do these findings mean?

- Our large-scale clinical data provide substantial evidence that the circadian variation in ischemic stroke progression observed in rodent models also modifies human ischemic stroke severity and course.
- These findings indicate that circadian time of onset may be an important additional variable to include in epidemiologic natural history studies and in treatment trials of neuroprotective and reperfusion agents for acute ischaemic stroke.
- Also, systematic basic, translational, and clinical science inquiry into the molecular, cellular, and systems chronobiology of acute brain ischemia is desirable and may unveil new pharmacologic targets for neurovascular therapeutic agent development.

**Comment 7**

Please temper the primacy claim in paragraph two of the introduction (‘To date, circadian effects on post-stroke END has never been investigated yet’) by adding ‘to our knowledge’.

**Reply 7**

We have added “to our knowledge” to the sentence.

**Comment 8**

Please provide the names of the institutional review boards that provided ethical approval.

**Reply 8**

Each of the 11 participating centers’ institutional review boards approved the study. In the revised manuscript, we provide the name of the representative institutional review board: Institutional Review Board of Dongguk University Ilsan Hospital, with which the principal investigator (Dong-Eog Kim) is affiliated.

**Comment 9**

Did your study have a prospective protocol or analysis plan? Please state this (either way) early in the Methods section.

a) If a prospective analysis plan (from your funding proposal, IRB or other ethics committee submission, study protocol, or other planning document written before analyzing the data) was used in designing the study, please include the relevant prospectively written document with your revised manuscript as a Supporting Information file to be published alongside your study, and cite it in the Methods section. A legend for this file should be included at the end of your manuscript.

b) If no such document exists, please make sure that the Methods section transparently describes when analyses were planned, and when/why any data-driven changes to analyses took place.

c) In either case, changes in the analysis—including those made in response to peer review comments—should be identified as such in the Methods section of the paper, with rationale.

**Reply 9**

We provide the prospective analysis plan as a Supporting Information file with a legend in the revised manuscript.

**Comment 10**

Citations should appear before punctuation throughout the manuscript.

**Reply 10**

Done.

**Comment 11**

Please define "lost to follow-up" as used in this study. Other reasons for exclusion should be defined.

**Reply 11**

In the revised manuscript, we define “lost to follow-up” and describe the exclusion criteria in more detail (highlighted), as follows:

Among these, 42903 were excluded sequentially according to the following reasons: (1) 3,375 patients refused to give research consent to being monitored for stroke outcomes; (2) 16,047 did not have the onset time information (9,922 wake-up stroke, 6,123 daytime-unwitnessed stroke, and 2 data missing); and (3) 23,481 arrived to the medical facility > 6 hours of onset. The remaining 17,461 patients with witnessed ischemic stroke (*n* = 14,890) or TIA (*n* = 2,481) constituted the study cohort. A total of 1,893 (12.8%) patients were lost to follow-up: did not complete hospital visit nor reply to phone call for outcome capture.

**Comment 12**

In Figures 1 and 2, please show the Y-axis beginning at zero. If this is not possible, please show a break in the axis.

**Reply 12**

We now show a break line in the revised Figures 1 and 2.

**Comment 13**

Please remove the ‘competing interests’ and ‘author contributions’ from the end of the main text – in the event of publication, this information will be published as metadata based on your responses to the submission form questions.

**Reply 13**

We have removed the ‘competing interests’ and ‘author contributions’ from the main text.

**Comment 14**

Thank you for providing your STROBE checklist. Please replace the page numbers with paragraph numbers per section (e.g. "Methods, paragraph 1"), since the page numbers of the final published paper may be different from the page numbers in the current manuscript.

**Reply 14**

Done. We thank you for all your comments.

**Reviewer #1**

**Comment 1**

My particular concern is the use of NIHSS to decimal places, which over-inflates the granularity of this measure. It is actually a categorical scale, so measures of 6.1 vs. 5.7 do not make any sense.  All analyses need to be re-done accordingly.

**Reply 1**

We thank the reviewer for pointing this out. In accordance with the reviewer’s request, all NIHSS-related analyses were re-done and the manuscript has been revised accordingly. Because the NIHSS has 43 score items (0-42), the proportional odds assumption is violated. Thus, we decided to categorize NIHSS scores into three groups. Due to the lack of consensus on the NIHSS score grouping, we used cut-off points of 0 – 1, 2 – 6, and ≥ 7, which allowed for each stratum to have a similar number of patients. Then, we could perform the multilevel mixed-effects ordered logistic regression analysis.

As shown in the Table below, compared with the 06:00–10:00 group, 18:00–22:00, 22:00–02:00, and 02:00–06:00 groups had a higher likelihood of more severe stroke. Specifically, the 02:00 – 06:00 group had a 45% higher likelihood of more severe stroke compared with the 06:00 – 10:00 group.

Applying parametric analysis to NIHSS distribution yield decimal values for groups, though not individuals, and removing the decimal values could reduce analytically relevant information. In addition, we may have to consider that many researchers and most readers assume that an NIHSS score, which is calculated by adding up the sums of ordinal categories, could be regarded as a continuous variable. Moreover, a group of stroke epidemiology researchers including a co-author of the present study (Jeffrey Saver) previously demonstrated that NIHSS score is a very strong discriminator of mortality risk, whether used as a continuous or categorical risk determinant (J. Am. Heart Assoc. 2012). In fact, a lot of papers, such as Lees et al. N Engl J Med 2006 and Goyal et al. Lancet 2016, provide mean NIHSS scores as decimal numbers to convey the sense of inter-group differences in stroke severity. Thus, we have retained the original NIHSS approach as our secondary analysis. However, given the reviewer’s concern, we provide the data as Supporting Information in the supplement of the revised manuscript.

Table for review. Unadjusted and adjusted associations between stroke onset time and stratified NIHSS scores

|  | Admission NIHSS | | | Adjusted common odds ratio |
| --- | --- | --- | --- | --- |
|  | 0 – 1 (n = 6,097) | 2 – 6 (n = 5,924) | ≥ 7 (n = 5,440) |  |
| 06:00–10:00 | 1,400 (35.7%) | 1,335 (34.0%) | 1,192 (30.4%) | Reference |
| 10:00–14:00 | 1,645 (35.4%) | 1,578 (34.0%) | 1,419 (30.6%) | 1.05 (0.96 to 1.15) |
| 14:00–18:00 | 1,361 (35.1%) | 1,272 (32.8%) | 1,247 (32.1%) | 1.09 (0.995 to 1.20) |
| 18:00–22:00 | 1,106 (34.0%) | 1,063 (33.7%) | 990 (31.3%) | 1.10 (1.01 to 1.23) |
| 22:00–02:00 | 360 (34.0%) | 393 (37.1%) | 307 (29.0%) | 1.22 (1.06 to 1.41) |
| 02:00–06:00 | 225 (28.4%) | 283 (35.7%) | 285 (35.9%) | 1.45 (1.23 to 1.69) |

Multivariable adjustment for age, sex, previous stroke, pre-stroke modified Rankin Scale score, admission National Institute of Health Stroke Scale score, hypertension, diabetes, hyperlipidemia, smoking, atrial fibrillation, pre-stroke antiplatelet use, pre-stroke statin use, revascularization, time from onset to hospital arrival, onset seasons and stroke subtype.

**Comment 2**

While 'night' vs. 'day' can be a simple comparison, the analysis for 4 hourly time periods should be undertaken as a p for trend rather than categories against a control group. A significant time epoch against control could purely be due to chance from multiple testing

**Reply 2**

We thank the reviewer for pointing this out. First, statistical tests for linear, quadratic, or cubic trend would not be appropriate for our data. Although sinusoidal regression analysis is a better method, it does not allow adjusting for covariates (such as demographic and clinical variables), which is very important in outcome research. Thus, we performed the logistic regression analysis. Second, we obtained p values by using a ‘single’ regression model, avoiding the multiple comparisons problem.

**Comment 3**

While adjustments have been made for hospital clustering and day of the week, there is also likely to be a seasonal component which also needs to be taken into account. Moreover, this also determines change in physiological variables, in particular systolic blood pressure but also blood glucose level, which may be particularly important on the ischemic penumbra and could be included in the models of baseline variables.

**Reply 3**

In accordance with the reviewer’s (and reviewer #3’s) comments, we have included seasons as a covariate in multivariable analyses: spring (March, April, and May), summer (June, July, and August), fall (September, October, and November), and winter (December, January, and February). We found that further adjustment for the seasonal component did not change the results.

**Comment 4**

Given variability in diagnosis (despite involvement of neurologists) and potential pathophysiology, I would like to see sensitivity analysis confined to acute ischemic stroke cases

**Reply 4**

We performed an additional sensitivity analysis after excluding 2,481 patients with transient ischemic attack. The association between stroke onset time and early neurological deterioration was maintained. In the revised manuscript, we have included the following:

Method

In addition, we performed a sensitivity analysis on the relationship between stroke onset time and early neurological deterioration after excluding a subset of patients with transient ischemic attack.

Results

In addition, the sensitivity analysis for the non-TIA patients showed that night-onset stroke patients had more frequent END compared with day-onset stroke patients (S4 Table).

**S4 Table. Multivariable associations between stroke onset time and early neurological deterioration: a sensitivity analysis after exclusion of patients with transient ischemic attack**

| **Stroke onset time** | **Unadjusted END, n (%)** | **Adjusted^*^ END, % (95% CI)** | **Adjusted risk difference**^†^**, % (95% CI)** | **p value** |
| --- | --- | --- | --- | --- |
| 06:00–18:00 | 1,527 (14.2%) | 14.7 (13.3 to 16.0) | Reference |  |
| 18:00–06:00 | 656 (15.6%) | 16.4 (14.7 to 18.0) | 1.7 (0.4 to 3.0) | 0.012 |

END=early neurological deterioration. CI=confidence interval. *Mixed-effects logistic regression with an interaction term between stroke onset time and weekdays (vs. weekend) was used. Multivariable adjustment for age, sex, pre-stroke modified Rankin Scale score, National Institute of Health Stroke Scale score, previous stroke, hypertension, diabetes, hyperlipidemia, atrial fibrillation, smoking, stroke subtype, time from onset to hospital arrival, pre-stroke antiplatelet use, season of stroke onset, and pre-stroke statin use. †Risk differences are for night-onset stroke relative to day-onset stroke.

**Comment 5**

Figure 1 should relate to 'frequency' rather than 'incidence'

**Reply 5**

Done.

**Reviewer #2**

**Comment 1**

Page 5 If some of the measurements are observations not planned in the protocol does this possibly introduce a bias?

**Reply 1**

All measurement data including stroke outcomes such as early neurological deterioration were prospectively collected by physicians and research nurses in the participating centers. The figure below shows the electronic CRF **(**<http://strokedb.or.kr/ecrf/>) that we have been using since 2010 June.

**Comment 2**

Page 5 It might be helpful to give the composition of the adjudication committee at least in terms of professional representation. I assume they did not adjudicate patients for whose clinical care they had been responsible?

**Reply 2**

We thank the reviewer for pointing this out. There are five core members in the adjudication committee: three neurologists (one of whom is a co-author of this paper; TH Park), two research nurses, and one research coordinator. We confirm that they do not adjudicate their own hospital patients’ data. This information has been added to the manuscript.

**Comment 3**

Page 6 The authors have used two different ways of dividing up the day. Categorising an essentially continuous variable wastes information (Altman and Royston, 2006; Royston et al., 2006) and leads to models which are often implausible as they predict the effect remaining flat within categories and then jumping to a new value at the category boundary. In this case is there an agreed convention for the 0600{1800 versus 1800{0600 split? There is plenty of data so I wonder whether even narrower categories than four{hourly would be better to show the form of relationship.

**Reply 3**

We thank the reviewer for pointing this out. The average sunrise and sunset times are 06:00 and 18:00, respectively (<https://www.worlddata.info/asia/south-korea/sunset.php>). Thus, we used 06:00~18:00 vs. 18:00~06:00 for the day-time and the night-time, respectively. We also used 4-hour time intervals, as were the cases for prior studies on the association between circadian rhythm and cardiovascular disease (Thosar et al. Arterioscler Thromb Vasc Biol. 2019; De Luca G, et al. Am Heart J. 2005; Fournier S et al. Eur Heart J Cardiovasc Pharmacother. 2018; Wu J, et al. Eur Heart J. 2019). In accordance with the reviewer’s request, we have tried using hourly intervals. The Figure below shows that further investigation with a larger sample size is required for a higher level of data granularity. In the revised manuscript, we acknowledge this as a limitation of our study, as follows:

Discussion

Fourth, further investigation with a larger sample size is required for a higher level of data granularity, which could demonstrate the association between stroke onset time and outcomes at relatively narrow time intervals.

**Figure for Reviewers.** Multivariable associations of stroke onset time (at 1-hour intervals) with admission National Institute Health Stroke Scale (NIHSS) scores, early neurological deterioration, and 3-month functional outcome. Dots and error bars indicate coefficients and their 95% confidence intervals, respectively.

**Comment 4**

Page 6 Functional outcome is an ordered categorical variable. Did the authors not consider using ordered logistic regression here?

Reply 4

As the reviewer nicely pointed out, we initially considered using ordered logistic regression analysis. However, due to the violation of the proportional odds assumption (chi-square = 875.07; p < 0.0001), we dichotomized patients. We now mention this in the revised manuscript.

**Comment 5**

Page 7 Some of the variables referred to here and displayed in Table 1 do not seem to me to be baseline characteristics. For example END, favourable outcome.

Reply 5

We thank the reviewer for pointing this out. We have removed the following dependent variables from Table 1 : admission NIHSS score, early neurological deterioration, and favorable outcome.

**Comment 6**

Page 9 Since the NIHSS score is presumably skewed why not just present
the exponentiated coefficient as a percentage increase with its confidence interval rather than two means?

Reply 6

As suggested by the reviewer (and the reviewer #1), we categorized NIHSS scores into three groups using cut-off points of 0 – 1, 2 – 6, and ≥ 7, which allowed performing the multilevel mixed-effects ordered logistic regression analysis. Thus, we now present common odds ratios and 95% confidence intervals in the main text of the revised manuscript.

**Comment 7**

Page 10 As someone with a limited knowledge of acute stroke care I struggle to understand why revascularized patients do so much worse.

Reply 7

The most common reason that patients do not undergo revascularization therapy is that they present with mild deficits, making good outcomes likely and the risks of endovascular intervention not acceptable. Accordingly, at baseline the -revascularization population has much more severe stroke than the non-revascularization population. Revascularization therapy improves their outcomes but not to the level of patients with initial mild deficits. This is mentioned in the Figure 1 legend of the revised manuscript.

**Comment 8**

Page 11, Figure 2 This is where I think finer categories might be helpful. I would be cautious anyway about comparing one category with another in statistical significance when it is really the shape which matters.

Reply 8

We thank the reviewer for pointing this out. Please see the Reply 3.

**Reviewer #3**

**Comment 1**

The authors confuse the difference between circadian (endogenous, and without any external time cue) and diurnal rhythms. Their data is in entrained conditions. Therefore, using the term circadian is wrong. This should be replaced by diurnal, time-of-day,… This is not a purely academic difference. Circadian refers to endogenou“ ("biologi”al") effects, whereas there are a number of exogenou“ ("circumstan”es") effects, too. This study cannot resolve this dichotomy and, as the authors partly acknowledge by listing some limitations, can only point to correlation but not causation.

Reply 1

We acknowledge that in the preclinical biological science literature the term circadian is reserved for endogenous rhythms. However, in the clinical, medical literature, the term circadian is used for both endogenous rhythms and for entrained rhythms. Numerically, “circadian” is employed far more often than “diurnal”: e.g PubMed search for NEJM+circadian yields 95 citations while PubMed search for NEJM+ diurnal yields 10 citations; PubMed search for JAMA+circadian yields 143 citations while PubMed search for JAMA+ diurnal yields 27 citations. Recent articles in the leading journals of medicine have employed circadian in this manner, e.g. : 1) NEJM: Circadian Mechanisms in Medicine. Allada R, Bass J.N Engl J Med. 2021;384:550-561; 2) Lancet Network: Leng Y, et al. Association Between Circadian Rhythms and Neurodegenerative Diseases. Lancet Neurol 2019;18:307-318; 3) JAMA Network: Leguia MG, et al. Seizure Cycles in Focal Epilepsy. JAMA Neurol 2021;78:454-463. As the circadian is the more common term in the medical literature, we have retained it. However, to address the reviewer’s concern we have added to the limitations section that we have followed the medical literature approach in use of the term for a phenomenon that would be termed diurnal in the preclinical literature. Finally, in keeping with the large literature, we have also kept the word “circadian time” in the title, but if the editors would prefer that we switch to diurnal, we would be happy to.

Discussion

Fifth, we have followed the medical literature approach in use of the term ‘circadian’ for a phenomenon that would be termed ‘diurnal’ in the preclinical literature.

**Comment 2**

It is therefore, in my view, not warranted to conclude in the Discussion that the "These associations appeared to be biologically driven." when pointing to the association between mice and human patients. Sleep between the species is very different, as are number of other characteristics important for stroke.

Reply 2

We thank the reviewer for pointing this out. For clarification, we have revised the sentence (highlighted).

However, the circadian effect on presenting stroke severity and END was not modified when stroke onset time was further stratified by weekdays vs weekend. Accordingly, the diurnal variations in severity and progression appear to be biologically driven, rather than due to variations in systems of care. Similar care quality on weekdays and weekends, is in line with recent reports [23, 24]. Moreover, after applying the SITS-MOST criteria of END, the independent association between stroke onset time and END was retained, which supports the robustness of our results. In addition, the incidence of END in our population (12.7%) was comparable with previous reports [25],[26].

**Comment 3**

Moreover, the authors do not comment on how there might be a biased introduced by their "unwitnessed stroke" exclusion criteria that makes them exclude about the same number of patients than they included. Maybe some time of day has a lower likelihood of strokes being witnessed. This should be discussed.

Reply 3

We thank the reviewer for pointing this out. We have added following sentences to Discussion.

Discussion

Wake-up stroke was shown to be associated with a greater initial severity and a worse functional outcome at 3 months, compared with wakefulness stroke (Jimenez-Conde J et al. J Neurol. 2007;254:782–8). In another study, daytime-unwitnessed stroke patients were more likely to receive reperfusion therapy due to earlier hospital arrival after symptom recognition, compared with wake-up stroke patients (Kim et al. Int J Stroke. 2016;11:212-20). Thus, the exclusion of unwitnessed stroke in the present study might have biased towards the null, potentially leading to an underestimation of the association between stroke onset time and outcomes.

**Comment 4**

The data is drawn throughout a period of about 9 years and there is nothing shown for seasonal effects nor discussed. This should be added to the caveats already mentioned (sleep, shift-work, chronotype).

Reply 4

We thank the reviewer for pointing this out. In accordance with the reviewer’s (and reviewer #1’s) comments, we have included seasons as a covariate in multivariable analyses: spring (March, April, and May), summer (June, July, and August), fall (September, October, and November), and winter (December, January, and February). We found that further adjustment for the seasonal component did not change the results.

**Comment 5**

In conclusion, I think this is an important study to make the medical community aware of daytime specific changes in disease and implications for treatment. There are a number of strengths here, but a few more caveats should be considered.

Reply 5

We thank the reviewer for the encouragement.

**All authors have read and approved the revised manuscript.** **We hope the revised manuscript will better meet the requirements of “PLOS Medicine” for publication. We thank you and the reviewers again for the thoughtful and constructive review.**

**Dong-Eog Kim MD, PhD**

Sincerely yours,

**Dong-Eog Kim, MD, PhD**

Professor, Department of Neurology, Dongguk University College of Medicine, Korea

Director, National Priority Research Center for Stroke, Korea

Director, Korean Brain-MRI Data Center, Korea
