# Supplementary material for: Association of ischemic stroke onset time with presenting severity, acute progression, and long-term outcome: A cohort study
Source: PLoS Med. 2022 Feb 4;19(2):e1003910. doi: 10.1371/journal.pmed.1003910 (PMC8815976; doi:10.1371/journal.pmed.1003910)
Supplement: Attachment — Submitted filename: Response_to_Editor.docx [file pmed.1003910.s014.docx]

**PMEDICINE-D-21-04039R2**

Association of circadian time of ischemic stroke onset with presenting severity, acute progression, and long-term outcome: A cohort study

**New Title**

Association of ischemic stroke onset time with presenting severity, acute progression, and long-term outcome: A cohort study’

2021-12-21

**Dear Editor**

We are very pleased to hear that this paper was accepted for publication in PLOS Medicine. We again thank you and reviewers of ‘PLOS Medicine’ for taking time to review our manuscript and giving us another chance to finalize the paper. We have revised the draft in accordance with your requests, highlighted in yellow.

**Requests from the Editor:**

**Comment 1**

Title: Please consider updating to ‘Association of ischemic stroke onset time with presenting severity, acute progression, and long-term outcome: A cohort study’.

**Reply 1**

We have changed the title, as suggested.

**Comment 2**

Data Availability Statement: Please provide a contact email address for the steering committee in addition to the URL already provided.

**Reply 2**

We have revised the Data Availability Statement as follows:

Data Availability Statement: The informed consent obtained from the study participants does not allow the data to be made freely available through any third party maintained public repository. However, data used for this submission can be made available upon reasonable request (stroke@stroke.or.kr) and the approval of the ‘Comprehensive Registry Collaboration for Stroke in Korea’ steering committee.

**Comment 3**

Author Summary: Please revise your author summary in line with our guidelines (<https://journals.plos.org/plosmedicine/s/revising-your-manuscript>). More specifically, the summary should be non-technical and suitable for non-expert readers (including scientists and non-scientists). Avoid the use of complex terminology wherever possible and please make bullet points single sentences. Assertions of causality should also be avoided given the observational nature of the study (refer instead to associations).

**Reply 3**

We have revised the author summary, as follows:

**Author Summary**

**Why was this study done?**

- It is known that there is a diurnal pattern in the distribution of ischemic stroke, suggesting an influence of circadian rhythms on stroke incidence. However, it is unclear whether circadian rhythms may also affect the clinical severity of stroke, and whether time-of-day of stroke occurrence may affect acute clinical worsening after stroke onset.

**What did the researchers do and find?**

- We performed a multicenter study of 17,461 consecutive patients with ischemic stroke to ask whether time-of-day of stroke occurrence affected initial clinical severity and progressive clinical worsening within the first 72 hours after onset.
- We found a circadian variation in clinical characteristics and course of ischemic stroke. Night-onset stroke patients were found to have greater clinical severity, and a higher likelihood of experiencing early neurologic worsening during the first 72 hours following symptom onset. These patients were also found to have a lower likelihood of favorable 3-month global disability outcome than day-onset stroke patients.
- Circadian variation patterns differed among ischemic stroke subtypes in a manner suggesting modulation by collateral flow availability: a) night-onset large artery atherosclerosis stroke patients showed more frequent early neurologic worsening and worse 3-month functional outcome, b) night-onset cardioembolic stroke patients had higher clinical severity and worse 3-month functional outcome, and c) small vessel disease patients did not show night-day differences.

**What do these findings mean?**

- Our large-scale clinical data demonstrate that there is a circadian variation in ischemic stroke progression and severity.
- Our clinical findings are similar to recent reports in preclinical rodent stroke models.
- These findings indicate that circadian time of stroke-onset may be an important factor to consider in future epidemiological studies and treatment trials for acute ischemic stroke.
- Further basic science and clinical investigation probing the chronobiologic mechanism of acute brain ischemia may help to identify new pharmacologic targets.

**Comment 4**

Line 111: Remove ‘sample-sized’.

**Reply 4**

Done.

**Comment 5**

Line 150: Rather than providing the name of one representative institutional review board, please provide all IRB names in the supporting information and cite here (e.g. S1 Supporting Information). See here for more details regarding supporting information files (https://journals.plos.org/plosmedicine/s/supporting-information).

**Reply 5**

We have added ‘S1 text: List of institutional review boards’.

**Comment 6**

Line 230: Please include the original (unchanged) prospective analysis plan and relocate the details of additional or revised analyses performed during the revision process to your manuscript methods instead.

**Reply 6**

Done.

**Comment 7**

Table 1: In the legend, please indicate the statistical test used to generate p-values. Please also present descriptive statistics consistently (e.g., seasonal data has ‘%’ in parentheses while other percentages do not).

**Reply 7**

Done.

**Comment 8**

Figure 1: Please indicate the statistical test used in the legend.

**Reply 8**

Done.

**Comment 9**

Table 2: Please provide the unadjusted comparisons as well as the adjusted comparisons.

**Reply 9**

Adding the unadjusted comparison data made the table too long and complicated. Thus, we have provided them as S3 table.

**Comment 10**

Lines 415-421: Please consider whether this content would be better placed in the limitations discussion in the paragraph below.

**Reply 10**

We thank you for pointing this out. We have moved the paragraph to the limitation part of Discussion.

**We deeply appreciate you helping us improve the manuscript.**

**Dong-Eog Kim MD, PhD**

Sincerely yours,

**Dong-Eog Kim, MD, PhD**

Professor, Department of Neurology, Dongguk University College of Medicine, Korea

Director, National Priority Research Center for Stroke

Director, Korean Brain-MRI Data Center
